# Light-induced crawling of crystals on a glass surface

**DOI:** 10.1038/ncomms8310

**Published:** 2015-06-18

**Authors:** Emi Uchida, Reiko Azumi, Yasuo Norikane

**Affiliations:** 1 Electronics and Photonics Research Institute, National Institute of Advanced Industrial Science and Technology (AIST), Central 5, Higashi 1-1-1, Tsukuba, Ibaraki 305-8565, Japan

## Abstract

Motion is an essential process for many living organisms and for artificial robots and machines. To date, creating self-propelled motion in nano-to-macroscopic-sized objects has been a challenging issue for scientists. Herein, we report the directional and continuous motion of crystals on a glass surface when irradiated simultaneously with two different wavelengths, using simple azobenzenes as a photoresponsive organic compound. The direction of the motion can be controlled by the position of the light sources, and the crystals can even climb vertical surfaces. The motion is driven by crystallization and melting at the front and rear edges of the crystal, respectively, via photochemical conversion between the crystal and liquid phases induced by the *trans*–*cis* isomerization of azobenzenes. This finding could lead to remote-controlled micrometre-sized vehicles and valves on solid substrates.

## Abstract (web summary)

Creating the motion of nanosized or microsized objects is essential for building robots at small scales. Here, Uchida *et al.* move photoresponsive organic crystals on a glass substrate using two lamps, which crystallize and melt the crystal front and the rear, respectively, to generate the moving force.

## Graphical abstract

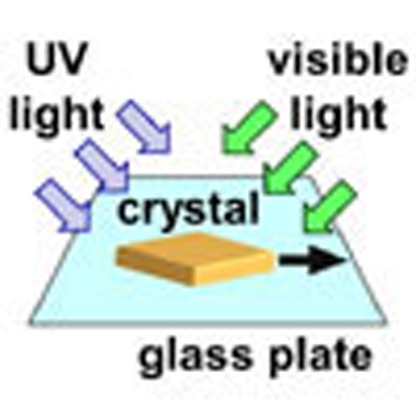

Motion is an integral process in living systems and is necessary for the performance of mechanical functions in artificial systems, such as robots and machines. Self-propelling motion of nano-to-macroscopic-sized objects has been of interest to scientists^1^,^2^,^3^,^4^,^5^,^6^,^7^. Target objects have been either liquids or solids, and physical or chemical energy has been used to obtain motion of these objects in fluids or on solid surfaces.

In liquid media or at liquid/air interfaces, self-propelling motion of objects has been observed when a chemical gradient existed around the objects. A well-known example is the camphor boat that moves on a liquid surface by asymmetric dissolution of camphor into the solution^7^,^8^,^9^. By releasing chemical substances pre-adsorbed in target objects that contain metal-organic frameworks^10^ or gels^11^, motion of these objects has been generated. Catalytic reactions, such as the decomposition of hydrogen peroxide, produce bubbles on the surface of a solid, resulting in the propulsion of solid boats or rods^1^,^2^,^3^,^4^,^5^,^6^,^7^,^12^,^13^. Liquid droplets also show self-propelling motion at liquid/air interfaces or in liquids that are driven by non-equilibrium chemical conditions^7^,^14^,^15^. As this motion is induced by a chemical gradient or non-equilibrium condition, the motion ceases when the gradient disappears or an equilibrium condition is established^7^.

Using light as an energy source, the motion of liquid droplets or solid objects has been demonstrated^7^. In these cases, light energy is used to generate a chemical gradient using a photochemical reaction, such as a photochromic reaction, or is converted to heat energy. On a liquid surface, for example, the motion of an oil droplet is obtained by generating a liquid/liquid interfacial gradient induced by the photochemical reaction of a photoresponsive surfactant^16^. In liquid crystals, the self-assembling nature of liquid crystal molecules has been utilized to induce directional motion of microparticles^17^,^18^ or rotation of sub-millimetre-sized glass rods^19^,^20^. A solid piece of polydimethylsiloxane, modified on one side by multiwall carbon nanotubes, moved on the surface of water when the modified surface was irradiated with a laser. In this case, the carbon nanotubes absorb light and convert it into heat energy, resulting in the generation of a surface tension gradient^21^. On the other hand, on solid surfaces it is quite challenging to induce translational motion of a liquid droplet or a solid object using light. To generate motion of liquid droplets, the solid surface has to be modified with photoresponsive molecules and precise control of the photoirradiation is required^22^,^23^,^24^. The motion of glassy solid objects on solid surfaces has been demonstrated; however, irradiation using a polarized laser was required^25^,^26^. Thin films of photochromic compounds, such as azobenzene (AB)-based polymers^27^,^28^ or small molecules^29^,^30^, show mass transport to form surface-relief gratings when irradiated with an interference pattern of coherent light. The range of the mass flow, however, is considered to be limited to the pitch of the interference pattern created by the coherent laser beams (generally up to several microns)^27^,^28^,^29^,^30^. However, photoinduced translational motion of crystals on untreated solid surfaces is unknown.

As described above, most studies have utilized the photoreactions of photochromic molecules to obtain photoresponsive self-propelling systems^16^,^17^,^18^,^19^,^20^,^22^,^23^,^24^,^25^,^26^. Organic photochromic compounds have also been utilized to induce various other mechanical motions of objects with light^31^. Molecular crystals^32^,^33^,^34^,^35^,^36^,^37^,^38^ and polymeric elastomers^39^,^40^,^41^,^42^ with photoreactive chromophores can exhibit bending^32^,^33^,^34^,^39^,^40^,^41^, twisting^43^, coiling^42^, expansion^35^ or even jumping^32^,^36^,^37^,^38^. In many cases, these mechanical motions involve the photochemical reactions of AB derivatives, which exhibit photoisomerization between *trans* and *cis* isomers on exposure to light^32^,^39^,^44^. Recently, by using the photoisomerization of AB, direct switching between solid and liquid phases was reported by our group and others^45^,^46^,^47^. This photoinduced phase transition has attracted interest not only in basic science^48^,^49^ but also in material applications such as photocontrollable adhesives^46^,^50^,^51^,^52^ and photoresists^53^.

Herein, we show the directional and continuous translational motion of crystals on a glass surface serendipitously observed in the course of our investigation on the photoinduced solid/liquid phase transition of AB derivatives. When irradiated simultaneously with two different wavelengths, crystals of simple ABs ‘crawl' on the surface and even ‘climb' vertical surfaces. The direction of the motion can be controlled by the position of the light sources. The motion proceeds without changing the crystal orientation, despite the large deformation of the crystal shape. The present method is particularly simple as crystals of a simple compound move on a bare glass surface using an light-emitting diode (LED) and a Hg lamp as light sources. Our results demonstrate that a bottom-up approach using simple small molecules can produce artificial non-living systems that move on solid surfaces.

## Results

### Translational motion of crystals with light irradiation

**Figure 1:**
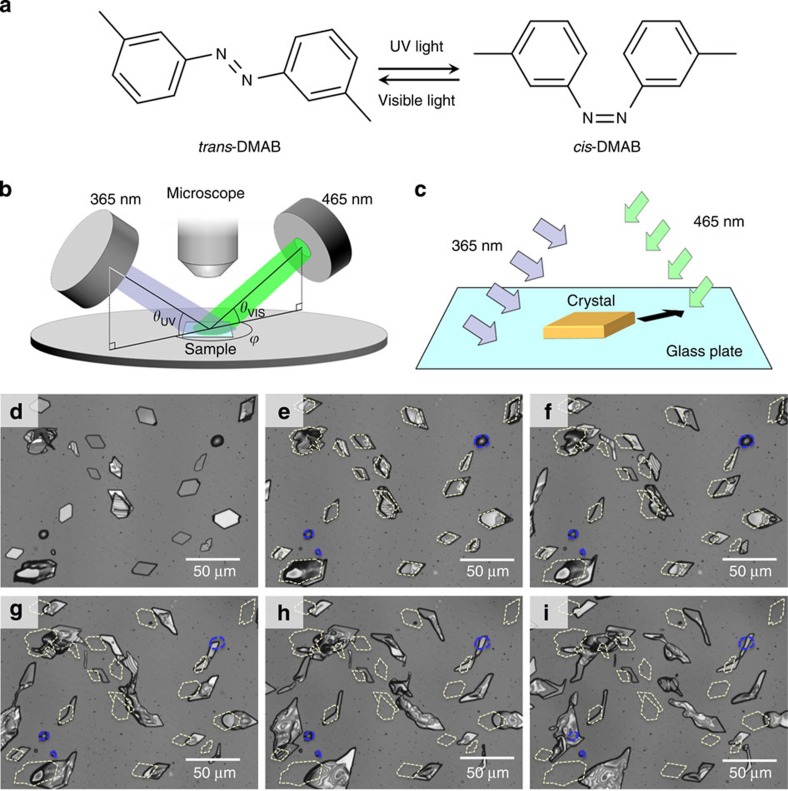
Photoinduced motion of crystals. (**a**) Molecular structure of 3,3′-dimethylazobenzene and its photoisomerization reaction scheme. (**b**) Schematic diagram of the experimental set-up. (**c**) Schematic of the crystal motion observed in this study. (**d**–**i**) Laser microscope images for the translational motion of DMAB crystals after irradiation for *t*=0 (**d**), 3 (**e**), 6 (**f**), 10 (**g**), 15 (**h**) and 20 min (**i**). The yellow and blue dashed lines represent the initial positions of the crystals and droplets, respectively. Irradiation conditions: *θ*_UV_=*θ*_VIS_=30°, *ϕ*=180°, intensities of UV and visible light are 200 and 100 mW cm^−2^, respectively. The UV and visible light are irradiated from the left and right side of the images, respectively.

Crystals of 3,3′-dimethylazobenzene (DMAB, Fig. 1a) placed on a glass substrate were irradiated with ultraviolet (UV) (365 nm) and visible (VIS) light (465 nm) using the experimental set-up shown in Fig. 1b (see Methods for details). The two wavelengths used for irradiation, 365 and 465 nm, correspond to the wavelengths that induce *trans*→*cis* and *cis*→*trans* isomerization, respectively (Supplementary Fig. 1). The irradiation resulted in a directional motion (Fig. 1c) of the crystals of DMAB, as shown in Fig. 1d–i (Supplementary Movie 1). The DMAB crystals seemed to move away from the UV light and towards the VIS light. Through the observation of a number of crystals, we found that the motion occurred regardless of whether the crystals were single crystalline, polycrystalline or a distorted shape (Fig. 1d–i). On average, the crystals moved ca. 15 μm in 10 min. However, the supercooled liquid droplets of DMAB did not show any translational motion (Fig. 1d–i, blue dashed circles). These results indicate that to obtain motion, the sample is required to be in the solid state.

It should be noted that the light sources used (high-pressure Hg lamp and LED) are neither coherent nor polarized, but are randomly oriented and incoherent. Furthermore, crystal motion proceeded with the light sources in a fixed position. It is notable that there is a no need for any special treatment, such as chemical modification^22^,^23^,^24^, spatial gradient^54^ or application of ratchet potential^55^, of the solid surface. The observed translational motion did not depend significantly on the condition of the glass surface. On glass surfaces with or without washing (including washing with acetone or UV/ozone cleaning), motion was observed. In addition, motion was observed on glass that had been previously treated with hexamethyldisilazane (HMDS), a reagent commonly used to generate hydrophobic glass and Si surfaces.

### Morphology and molecular orientation of crystals

**Figure 2:**
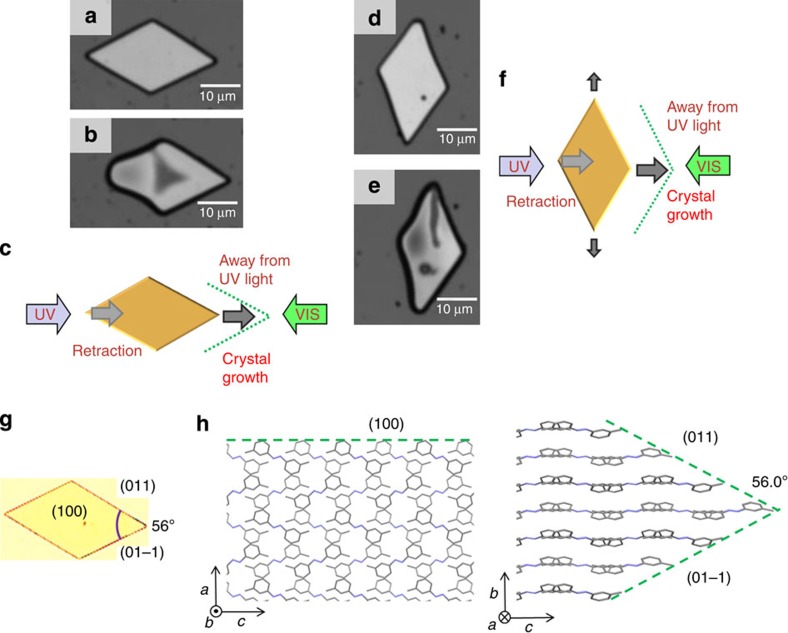
Crystal orientation and deformation. (**a–f**) Motion of the crystals when the longer diagonal axis of the crystal is (**a–c**) parallel and (**d–f**) perpendicular to the direction of irradiation. Laser microscope images of DMAB crystals before (**a**,**d**) and after (**b**,**e**) irradiation for 2 min. Slight deformations of the crystals are observed during the motion. Note that the crystals moved ca. 3 μm. Irradiation conditions: *θ*_UV_=*θ*_VIS_=30°, *ϕ*=180°, intensities of UV and visible light are 200 and 100 mW cm^−2^, respectively. (**c**,**f**) Schematic diagrams of the motion and the direction of light. (**g**) Optical photomicrograph of the crystal showing the crystal faces. (**h**) Schematic representation showing the molecular packing and crystal faces based on X-ray diffraction experiments.

The direction of crystal orientation was maintained despite the high deformity observed in the crystal shape during motion. For example, a parallelogram-shaped plate crystal was irradiated from the direction parallel to the longer diagonal axis of the crystal (Fig. 2a–c). In this case, the front edge of the crystal grew, whereas the rear edge shrank, and the whole crystal moved away from the UV light source. On the other hand, when the irradiation was perpendicular to the longer diagonal axis of the crystal, the shape of the crystal changed with the elongation observed along the longer diagonal axis (Fig. 2d–f). Notably, the angles between the edges of the crystal shown in Fig. 2b,e are ca. 56° and 124°, which are the same as the angles of the corners in the original crystal (Fig. 2a,d). The three-dimensional image observed using a laser microscope (Supplementary Movie 2) showed that the initial thickness of the crystals was 1–4 μm, and the crystals became thinner during the motion, especially at the front edge. Observations by polarizing optical microscopy showed that the direction of optical anisotropy of the crystal was maintained during the movement (Supplementary Movie 3), indicating that the crystal moves without changing its molecular orientation. These experiments indicated that the motion is likely caused by retraction of the rear edge and a crystal-growing process at the front edge. To obtain information about the surface morphology of the crystals, observations were carried out using atomic force microscopy (AFM). It was not possible to perform measurements *in situ* during the motion of crystals, owing to the limitations of the instrumentation used. Instead, we obtained AFM images before and after irradiation (Supplementary Fig. 2). Although we observed no significant change in the surface morphology before and after irradiation, the shape change of the crystals was similar to that observed by laser microscopy.

Single-crystal X-ray diffraction analyses were carried out to determine the molecular orientation and crystal faces. The observed crystal structure of DMAB was orthorhombic and was consistent with the literature^56^. The crystal faces of the parallelogram-shaped crystal were determined to be (1 0 0), (0 1 1) and (0 1 −1) for the top and side faces, respectively, as shown in Fig. 2g. The calculated angle between the two side faces ((0 1 1) and (0 1 −1)) is 56.02°, which is in good agreement with the measured angle (ca. 56°) of the crystal. The direction of the molecular orientation along the *c* axis (Fig. 2h) is in accordance with the polarizing optical microscopy observation that one of the optic axes is along the longer diagonal axis of the crystal.

### Effect of light intensity on the translational motion of crystals

**Figure 3:**
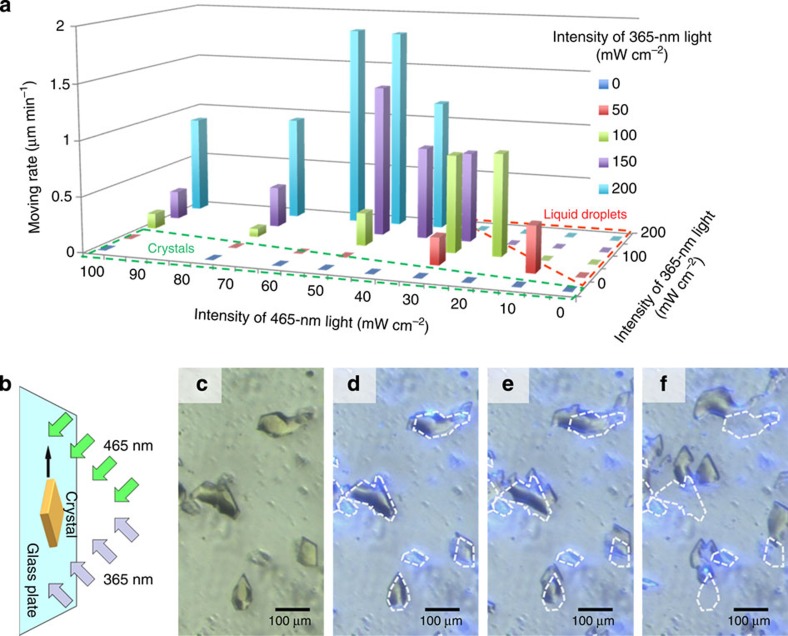
Crystal motion observed under various conditions. (**a**) Dependence of translational motion on light intensity when angles of irradiation are fixed (*θ*_UV_=*θ*_VIS_=45°, *ϕ*=180°). The motion of the crystals was statistically analysed and the average velocities are plotted (see Methods for details). (**b**) Schematic representation showing the vertical motion of a crystal. (**c**–**f**) Optical photomicrographs of DMAB crystals moving in a vertical direction following irradiation for *t*=0 (**c**), 2.5 (**d**), 5 (**e**) and 10 min (**f**). The dashed lines represent the initial positions of the crystals. Irradiation conditions: *θ*_UV_=*θ*_VIS_=45°, *ϕ*=180°, intensities of UV and visible light are 200 and 60 mW cm^−2^, respectively.

To understand the effect of the light intensity, the motion was observed by varying the intensity of the two light sources at the fixed angles of *θ*_UV_=*θ*_VIS_=45°, *ϕ*=180° (Fig. 1b). Irradiation of the crystals with only UV light liquefied the crystal, but the droplet formed did not move. Irradiation with only VIS light at different angles did not cause any movement or liquefaction; the crystals stayed at the original position and kept the same shape. Therefore, to obtain motion, the sample must be irradiated simultaneously with two light sources. The motion of the crystals was analysed statistically and the velocity was plotted (Fig. 3a; Supplementary Fig. 3), revealing that the relative intensity of the two light sources is important. When the intensity of the UV light is too strong, relative to that of the VIS light (Fig. 3a, red dashed line), the crystals melt to droplets, which do not move on the surface, as described earlier. On the other hand, when the UV light is relatively weak (Fig. 3a, green dashed line), the crystals stay at their original positions without changing their morphology. The optimum conditions for motion were found at UV and VIS light intensities of 200 and 50–60 mW cm^−2^, respectively, and the average velocity of the crystal motion was 2.0 μm min^−1^.

### Effect of irradiation angle on the translational motion of crystals

To determine the dependence of the motion on the angles of the irradiated light, the angles of the light sources (*θ*_UV_ and *θ*_VIS_) were scanned while fixing the intensities of the UV and the VIS light at 200 and 60 mW cm^−2^, respectively. In our experimental set-up, *θ*_UV_ and *θ*_VIS_ were varied in the range of 25–45° and 20–45°, respectively. The average velocity depends more on the angle of the UV light than on that of the VIS light, with a higher velocity obtained at higher angles of *θ*_UV_ (Supplementary Fig. 4).

The effect of the angle between the two light sources (*ϕ*) on the crystal motion was investigated at fixed *θ*_UV_ and *θ*_VIS_ (30°). When *ϕ*=135°, the crystals moved away from the UV light source (Supplementary Movie 4), indicating that the direction of the motion is dominated by the direction of the UV light.

### Motion on a vertical surface

To our surprise, the crystals climbed vertically on a wall of glass; even when the entire experimental set-up was turned sideways 90°, motion was still observed (Fig. 3b–f; Supplementary Fig. 5; Supplementary Movie 5). This result clearly shows that the translocation phenomenon observed in this study is not caused by gravity or other artefacts, but is a result of photoirradiation.

## Discussion

On the basis of the above observations, we propose that the driving mechanism of motion in DMAB crystals is the non-equilibrium condition established by light irradiation from two different directions. The entire sample is uniformly illuminated by two light sources and the light intensity seems to be virtually constant, because the distances to the light sources are substantially larger than the distance between the front and rear edges of a single crystal. However, for the lateral face of the crystal, the intensity ratio between UV and VIS light is different at the front and rear edges.

We analysed the isomer content of the sample and found that irradiation with UV light facilitates isomerization to the *cis*-isomer and induces liquefaction. On the other hand, irradiation with VIS light causes isomerization to the *trans*-isomer and results in crystallization. Notably, isomerization mainly proceeds on the surface of the crystal and rarely occurs inside the crystal, owing to steric hindrance in the crystal lattice^57^,^58^,^59^. The liquefied portion of the crystal consists of a mixture of *trans-* and *cis*-isomers. Here, the isomer ratio at a certain position is dependent on the location, because the intensity ratio between UV and VIS light differs owing to the three-dimensional shape of the crystal.

Thus, the crystal itself consists almost entirely of *trans*-isomers and an adsorption–desorption process of the *trans-*isomer should occur at the crystal/liquid interface. In addition, the speed of adsorption and desorption must be different at different concentrations of the *trans*-isomer in the liquefied portion. Therefore, non-equilibrium conditions occur at the front and rear edges of the crystal, leading to crystal growth and crystal melting. Using the optimized conditions, the motion proceeded continuously; this result is in sharp contrast to that of small particles^18^ in fluidic systems where the motion ceased upon prolonged irradiation.

To confirm that the crystal-growing process on the glass surface is involved in the translational motion, the photoirradiation was carried out from the back side, that is, from the face where the crystals contact the glass. When the back side of the sample was irradiated, crystal motion was not clearly observed, although the morphology of the crystals was changed (Supplementary Fig. 6a; Supplementary Movie 7). In this case, the crystal surface at the crystal/glass interface was melted by the photoirradiation and the crystals could not make firm contact with the glass surface.

We also attempted to observe motion using AB crystals. Motion was not observed when AB crystals were irradiated at room temperature. However, the crystal started to move when it was heated to 50 °C (Supplementary Movie 6). This result is in good agreement with the observation that the AB crystals do not exhibit photoinduced liquefaction at room temperature but do at 50 °C. Therefore, it can be anticipated that the translational motion observed in this study can be applied to other molecular systems that show reversible crystal-to-liquid phase transitions utilizing AB^53^ and other photochromic materials.

The crystal translocation observed in this study is reminiscent of the amoeboid motion of living organisms such as migrating cells^60^. When a cell moves on a surface, the process involves cell polarization, protrusion and adhesion and rear retraction^60^. In this study, polarization occurred through light irradiation and crystal orientation. Protrusion can be regarded as crystallization at the front edge, and rear retraction as melting of the crystal at the rear edge.

In summary, photoinduced directional motion of crystals on a glass surface was demonstrated for a simple organic compound using light irradiation at two different wavelengths (365 and 465 nm) from different directions at room temperature. The crystals moved away from the UV light source, and the molecular orientation of the initial crystal was maintained. Our results demonstrate that a bottom-up approach using simple small molecules can produce artificial non-living systems with motion comparable to the amoeboid or crawling motion of living organisms. This finding has applications in remote-controlled micrometre-sized vehicles and valves on solid substrates.

## Methods

### Materials and methods

DMAB and AB were purchased from Tokyo Chemical Industry Co., Ltd and purified by silica gel column chromatography. Subsequent recrystallization from methanol gave *trans*-isomers (*trans*-DMAB and *trans*-AB). The *cis*-isomer of DMAB (*cis*-DMAB) was prepared by crystallization from a hexane solution of DMAB that was at the photostationary state obtained by irradiation with UV light (365 nm). Hexamethyldisilazane was purchased from Sigma-Aldrich and used as received. Cover glasses (Matsunami Glass Ind., Ltd, square microscope cover glass No. 1, 18 × 18 mm, thickness: 0.12–0.17 mm) were used as the glass substrate for the optical observations. The transmittance of the cover glass was 90.5% at 365 nm, and 91.5% at 465 nm. This results show that the light sufficiently penetrates through.

UV–VIS absorption spectra were measured using a Shimadzu UV3100S spectrophotometer. Differential scanning calorimetry (DSC) thermograms were obtained using a SII Nanotechnology DSC6100. DSC thremograms of *trans-* and *cis*-DMAB are shown in Supplementary Fig. 7. Photoirradiation experiments were carried out at 365 nm (10 nm half bandwidth) using a 250-W high-pressure Hg lamp (Asahi Spectra Inc., REX-250) equipped with a bandpass filter (Asahi Spectra Co., Ltd, LX0365) and cold mirror module and at 465 nm using an LED lamp (Brainvision Inc. LEX2). The two wavelengths used for irradiation, 365 and 465 nm, correspond to the wavelengths that induce *trans*–*cis* and *cis*–*trans* isomerization, respectively (Supplementary Fig. 1). For the experiments on the translational motion of the crystals on a glass substrate, photoirradiation was carried out with UV (365 nm) and VIS light (465 nm) using the experimental set-up shown in Fig. 1b under ambient atmosphere. A UV light source (365 nm, high-pressure Hg lamp) is positioned pointing towards the sample at an angle, while a VIS light source (465 nm, LED) is placed in a different location and directed towards the sample. The entire sample area is irradiated by these light sources.

The motion of the crystals and three-dimensional images were obtained using a Keyence VK-X100 laser microscope with a laser wavelength of 658 nm, which is outside the range where the AB samples absorb. Light intensity and light angle dependence studies were observed with an OLYMPUS BX51 microscope equipped with a digital camera. The ‘vertical' motion of crystals was observed with a Nikon SMZ-1 stereo microscope equipped with a digital camera using the experimental set-up shown in Supplementary Fig. 5. The light intensity was monitored using a Newport 1917-R optical power meter with an 818-ST-UV photodetector. The sample temperature was controlled by a heating/cooling stage (Linkam 10033L). For photoirradiation experiments, with the exception of the vertical experiments, the sample temperature was kept at 25 °C for DMAB and 50 °C for AB using a heating/cooling stage. The vertical experiments were carried out at ambient temperature (ca. 20 °C). AFM images were obtained with an AFM unit (SPA400/SPI4000, SII NanoTechnology) operated in tapping mode in ambient atmosphere.

Single-crystal X-ray diffraction experiments were performed on a Bruker AXS APEX2 diffractometer with Mo Kα radiation. All crystal structures were solved by direct methods and refined using a full-matrix least-squares analysis (SHELXL-97). The crystal data are summarized in Supplementary Fig. 8. The determination of the crystal faces was performed on a Bruker AXS APEX2 for the (0 1 1) and (0 1 −1) faces, and a Rigaku RU-300 (Cu Kα, 40 kV, 200 mA, in the 2*θ*−*θ* scan mode with 0.01° step) for the (1 0 0) face.

### Sample preparation

To obtain similarly sized single crystals on a glass substrate reproducibly, we prepared crystals using the following method. Initially, DMAB sandwiched between two cover glasses was heated above the melting temperature. The sample was then cooled to room temperature. After formation of crystals, the two glass plates were separated so that a glass partially covered with crystals was obtained. The irradiation of the sample with UV light to partially melt the crystals via *trans*-to-*cis* isomerization (photoinduced phase transition, Supplementary Fig. 9) was observed using an optical microscope. The irradiation was ceased before complete liquefaction of the crystals so that the remaining crystals could be used as seeds (without seeds, it was difficult to obtain small-sized crystals). Then the partially melted sample was irradiated with VIS light to induce *cis*-to-*trans* isomerization and the crystals grew from the seeds. Finally, to prevent sublimation of crystals, the whole sample was covered with another clean glass using a spacer (scotch tape). The prepared crystals contained the *trans*-DMAB isomer.

### Data analyses of translational motion of crystals

In each experiment for the translational motion, the travel distances of the crystals in the whole field of view were measured. The distance was defined as the lateral distance moved by the front edge of the crystal, as shown in Supplementary Fig. 10. We measured the moving distance of all crystals in a field of view, except for the cases when more than two crystals merged into one. For the experiments on the intensity dependence and angle dependence, the motion was statistically analysed, and each measurement contained data from 26–84 crystals. The moving distances of crystals are plotted as dot plots shown in Supplementary Figs 3 and 4.

## Additional information

**How to cite this article**: Uchida, E. *et al.* Light-induced crawling of crystals on a glass surface. *Nat. Commun.* 6:7310 doi: 10.1038/ncomms8310 (2015).

**Accession codes**: Atomic coordinates for the reported crystal structure has been deposited in the Cambridge Structural Database with registry number CCDC 1035342. Readers are welcome to comment on the online version of the paper.

## Supplementary Material

Supplementary Figures and References Supplementary Figures 1-10 and Supplementary References

Supplementary Movie 1 Motion of DMAB crystals observed by laser microscope. The scale bar corresponds to 50 μm. Original photographs are shown in Figure 1. Fast forward speed at 100×real time.

Supplementary Movie 2 Motion of DMAB crystals in 3D observed by laser microscope. The corresponding 2D images are shown in Figure 1 and Supplementary Movie 1. Fast forward speed at 100×real time.

Supplementary Movie 3 Motion of DMAB crystals observed by polarizing optical microscope at crossed polarizer orientation. The initial and final frames were recorded at perpendicular polarizer orientation. The scale bar corresponds to 50 μm. Fast forward speed at 100×real time.

Supplementary Movie 4 Motion of DMAB crystals at f = 135° observed by optical microscope. The scale bar corresponds to 50 μm. Fast forward speed at 100×real time.

Supplementary Movie 5 Motion of DMAB crystals in a vertical direction observed by stereo microscope. The scale bar corresponds to 200 μm. Experimental setup is shown in Supplementary Figure 5. Fast forward speed at 100×real time.

Supplementary Movie 6 DMAB crystals observed by optical microscope when the back side of the sample was irradiated. Experimental setup is shown in Supplementary Figure 6a. The scale bar corresponds to 50 μm. Fast forward speed at 100×real time.

Supplementary Movie 7 Motion of AB crystals at 50 °C observed by optical microscope. The scale bar corresponds to 50 μm. Fast forward speed at 100×real time.
