# Supplementary material for: Light-induced crawling of crystals on a glass surface
Source: Nat Commun. 2015 Jun 18;6:7310. doi: 10.1038/ncomms8310 (PMC4557305; doi:10.1038/ncomms8310)
Supplement: Supplementary Figures and References — Supplementary Figures 1-10 and Supplementary References [file ncomms8310-s1.pdf]

1. UV-vis absorption spectroscopy

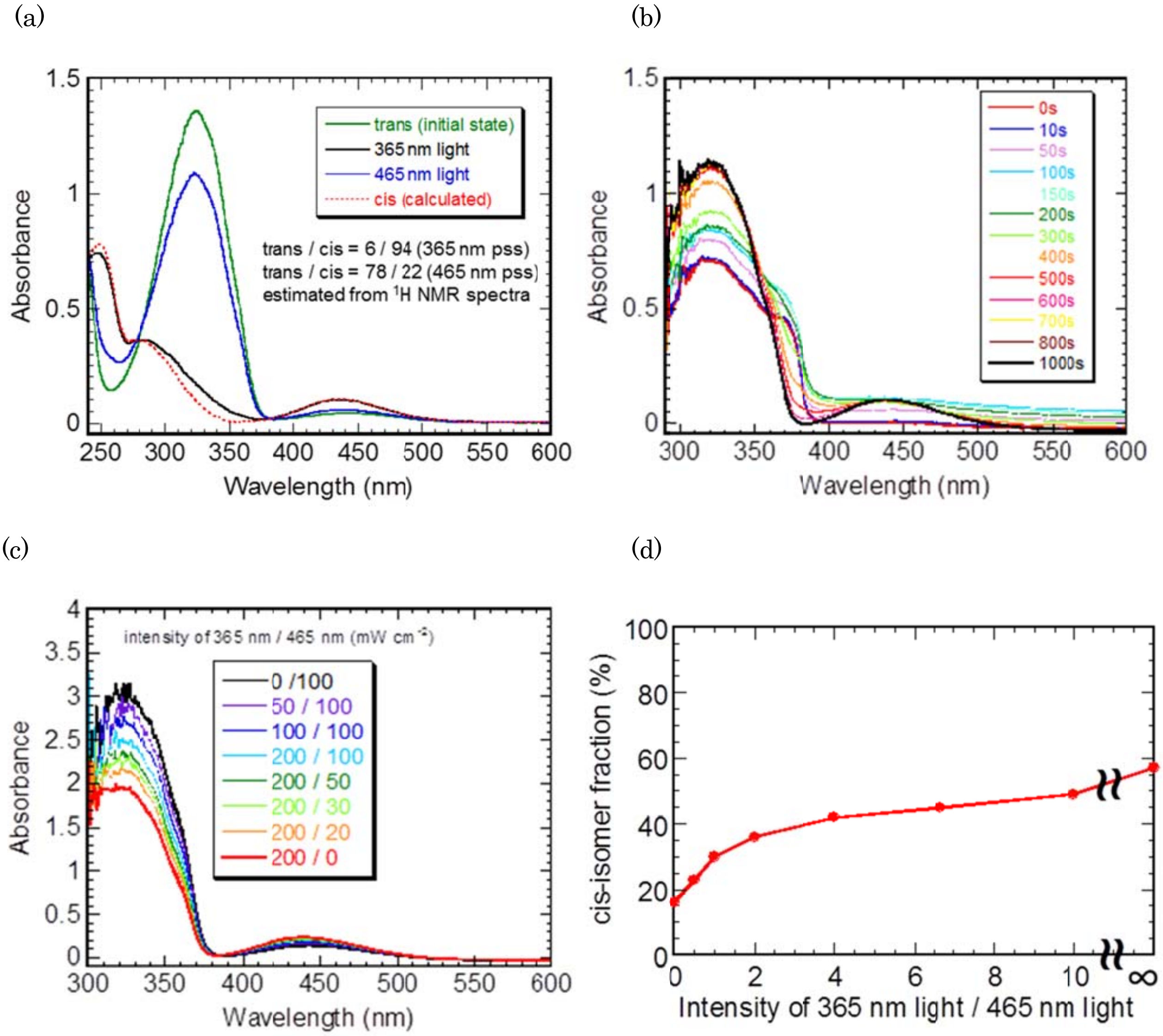

Supplementary Figure 1 Absorption spectra of DMAB (a) in chloroform and (b) in a film of *trans*-DMAB upon irradiation of 365 nm, and (c) in a film of DMAB at the photostationary state when irradiated simultaneously with 365 and 465 nm. (d) The fraction of cis-isomer estimated from the absorption spectra (c). (a) In solution the *cis* isomer is dominant (94% estimated from ^{1}H NMR spectra) upon irradiation at 365 nm, whereas the *trans* isomer is dominant (78%) upon irradiation at 465 nm. (b) The crystalline film state of *trans*-DMAB exhibits photoisomerization upon irradiation at 365 nm. (c) (d) The isomer ratio varies when the film is irradiated simultaneously at 365 and 465 nm.

2. AFM images of crystals before and after photoinduced translational motion

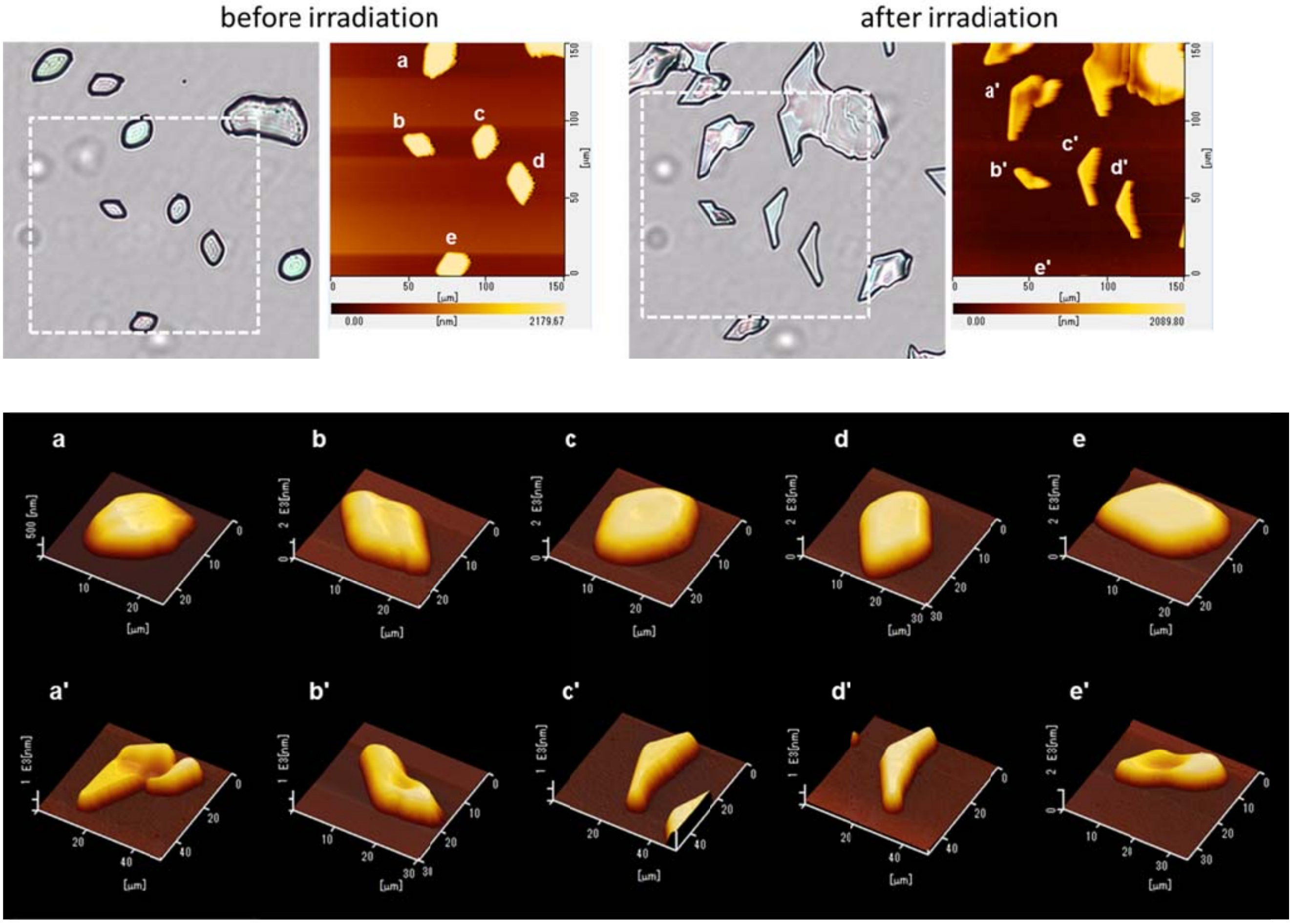

Supplementary Figure 2 AFM images of DMAB crystals before and after irradiation to induce translational motion. Optical microscope images are also shown.

3. Light intensity dependence

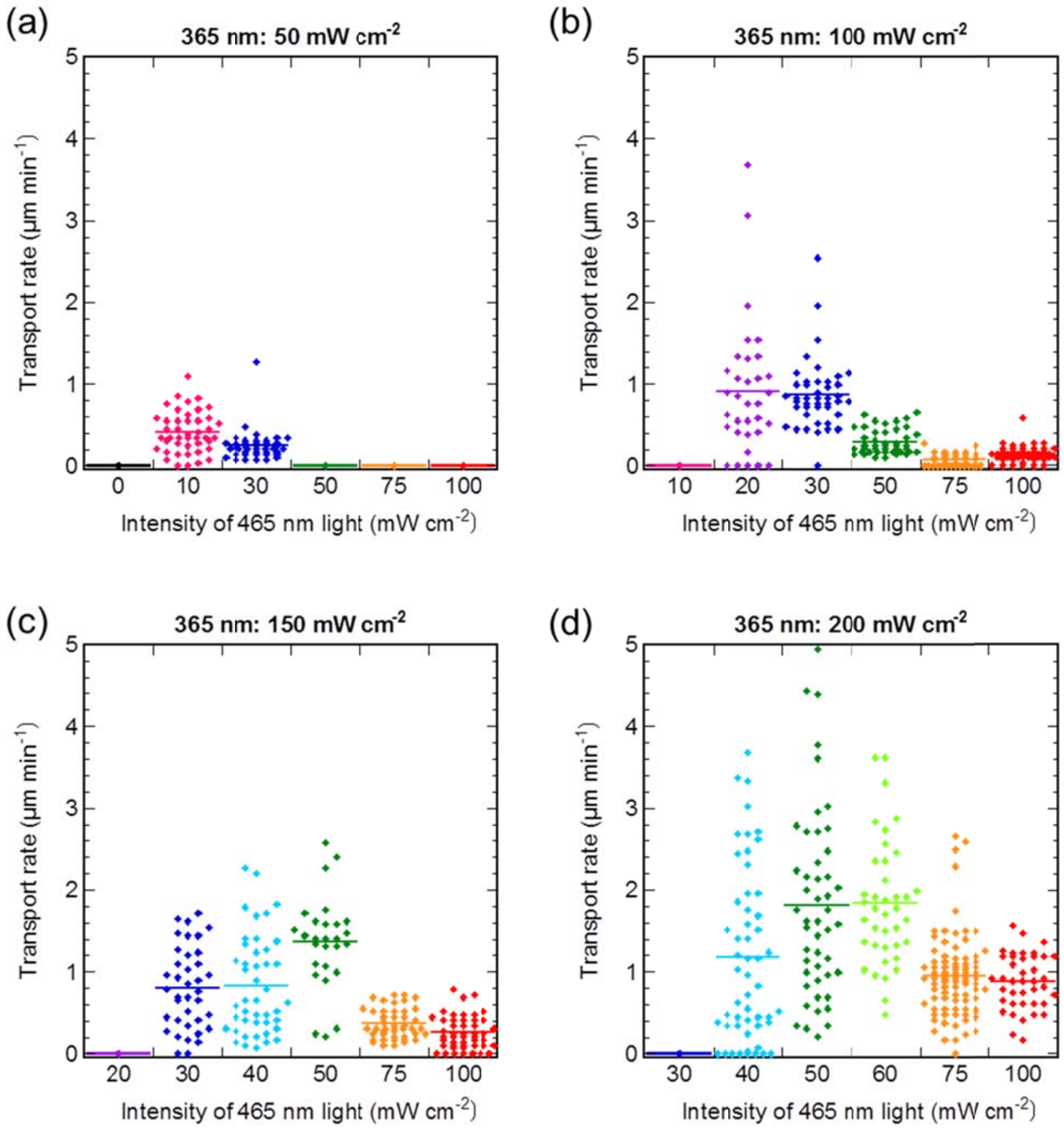

Supplementary Figure 3 Dot plots of the light intensity dependence of the motion of DMAB crystals. Horizontal lines indicate average values. Each dot represents each single crystal. Only a single dot is shown in the case when no motion is observed.

4. Angle dependence

(a)

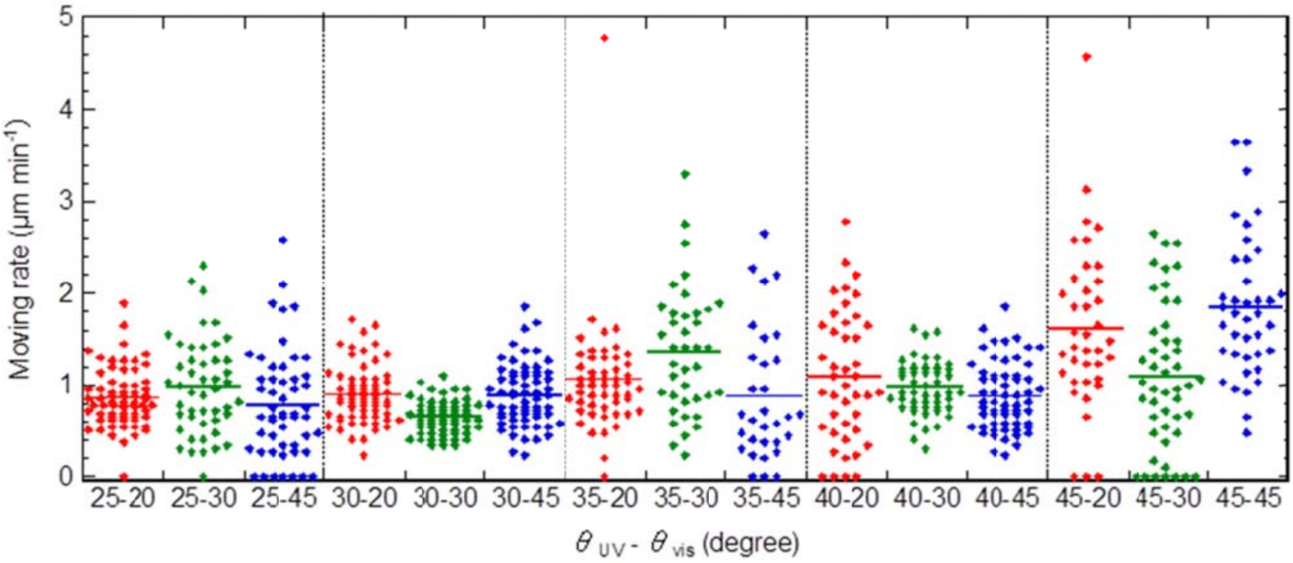

(b)

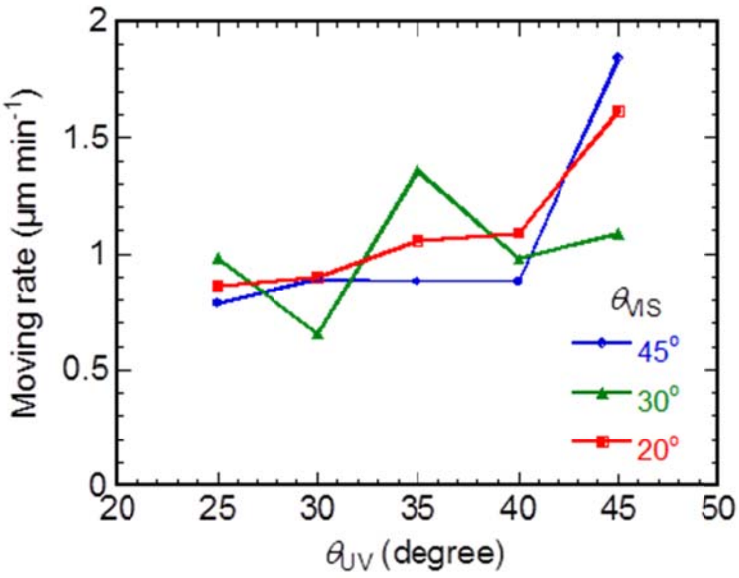

Supplementary Figure 4 (a) Dot plots and (b) average plots of the angle dependence of the motion of DMAB crystals. Angles of light source (θ_{UV} and θ_{VIS}) are varied when the intensity of the UV and visible light sources are fixed at 200 and 60 mW cm^{-2}, respectively. The motion of the crystals was statistically analysed and the average rates are plotted. Horizontal lines in (a) indicate average values. Each dot represents each single crystal.

5. Vertical motion of crystals

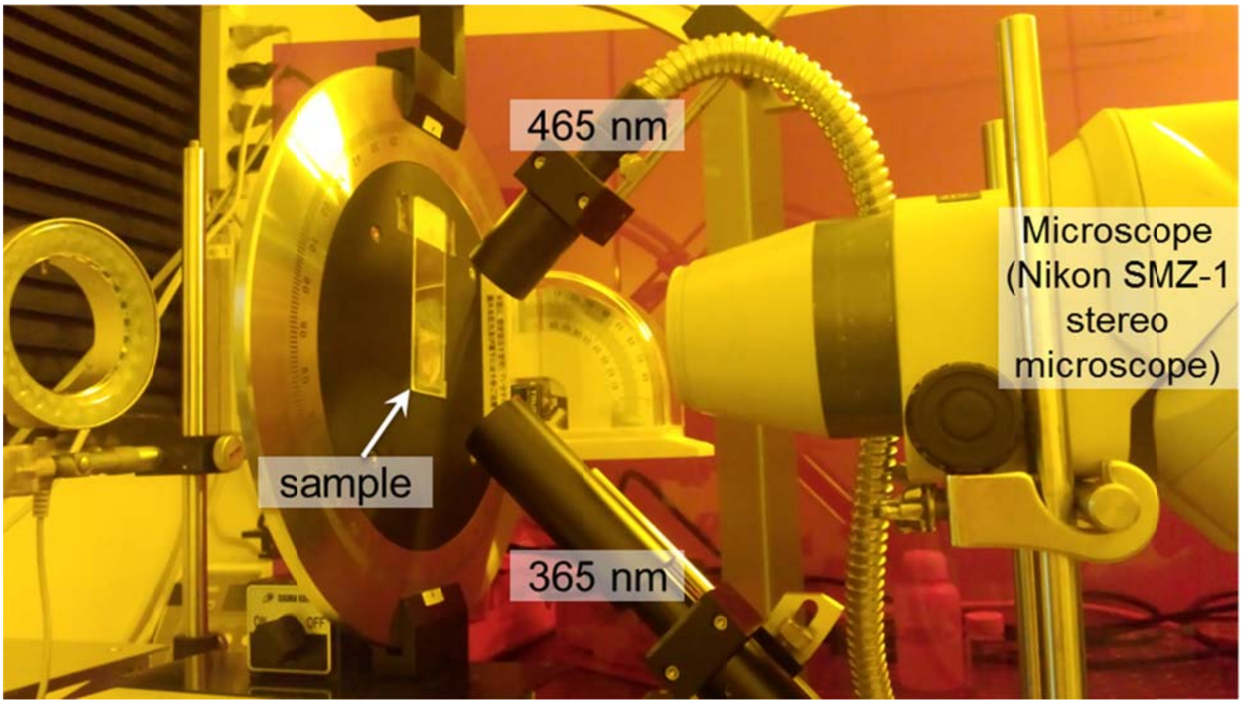

Supplementary Figure 5 Photograph showing the experimental setup for the observation of vertical motion.

6. Photoirradiation from back side of sample

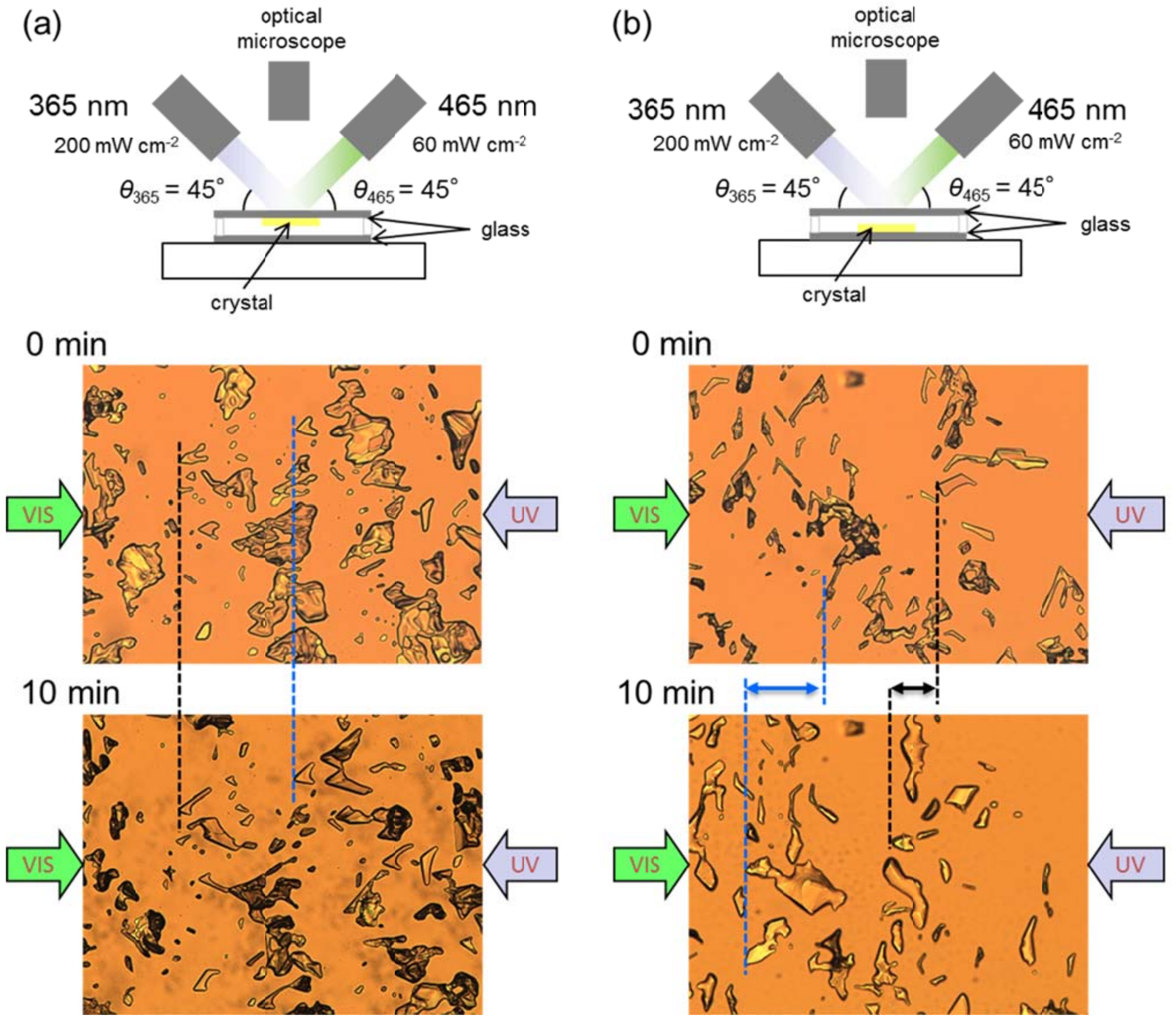

Supplementary Figure 6 Schematic representation of the experimental setups, and optical photomicrographs of DMAB crystals when irradiated (a) from back side and (b) front side of sample. The irradiation time is 10 min. Note that the pictures are reversed images and the irradiation direction has been reversed compared with the experimental setup because an optical microscope was used in this experiment. (a) The motion was not observed. (b) The motion was observed.

7. Differential scanning calorimetry (DSC)

(a) (b)

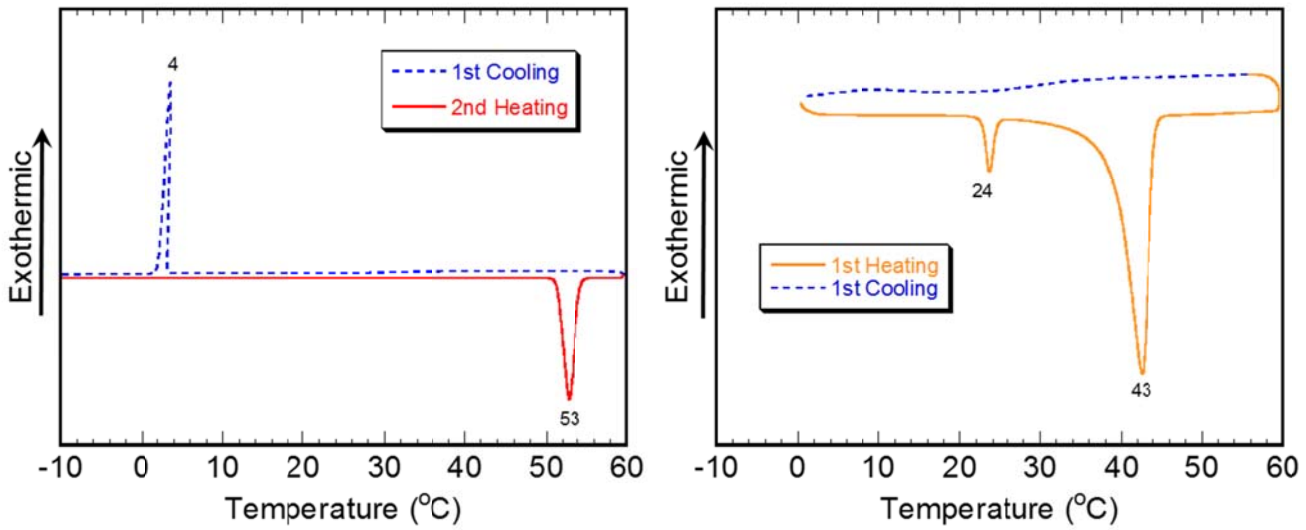

Supplementary Figure 7 Differential scanning calorimetry (DSC) thermograms of (a) *trans*-DMAB for the first cooling and second heating cycle and (b) *cis*-DMAB for the first heating and first cooling cycle at a scan rate of 5 K min^{-1}. (a) The endothermic peak at 53 °C indicates that the melting point of *trans*-DMAB is 53 °C, which is in good agreement with the reported value (53 °C) [1]. The exothermic peak at 4 °C indicates that *trans*-DMAB shows supercooled liquid state at room temperature. (b) The endothermic peak at 43 °C corresponds to the melting point of *cis*-DMAB, which is in fair agreement with the reported value of 46 °C[2].

8. Single crystal X-ray crystallography

Crystal data for *trans*-DMAB :

$C_{14}H_{14}N_2$, M = 210.27, orthorhombic, *Pbca*, a = 11.7185(8) Å, b = 7.3487(5) Å, c = 13.8144(9) Å, V = 1189.64(14) Å^3, Z = 4, D_{calc} = 1.174 gcm^{-3}, crystal size 0.40 × 0.20 × 0.05 mm^3. Diffraction data were collected at 183 K with Mo Kα radiation. R = 0.0427, w$R2$ = 0.1382, and S = 1.043.

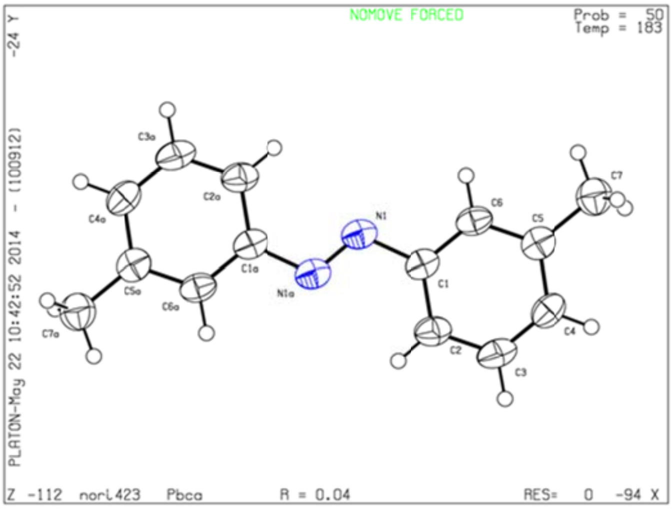

Supplementary Figure 8 X-ray crystallographic data for *trans*-DMAB.

9. Photoinduced crystal-liquid phase transition of 3,3’-dimethylazobenzene

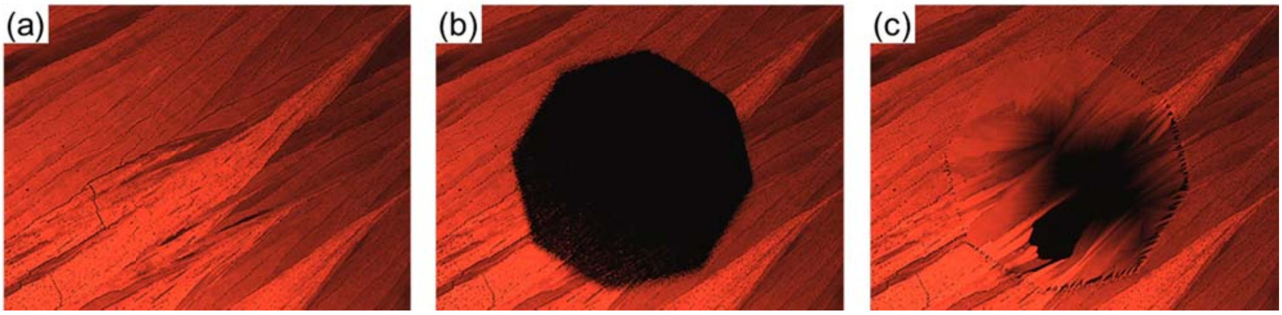

Supplementary Figure 9 Polarizing optical photomicrographs of a thin film of *trans*-DMAB crystals (a) before irradiation, (b) after the irradiation at 365 nm, and (c) after subsequent irradiation of 436 nm.

10. Data analyses of translational motion of crystals

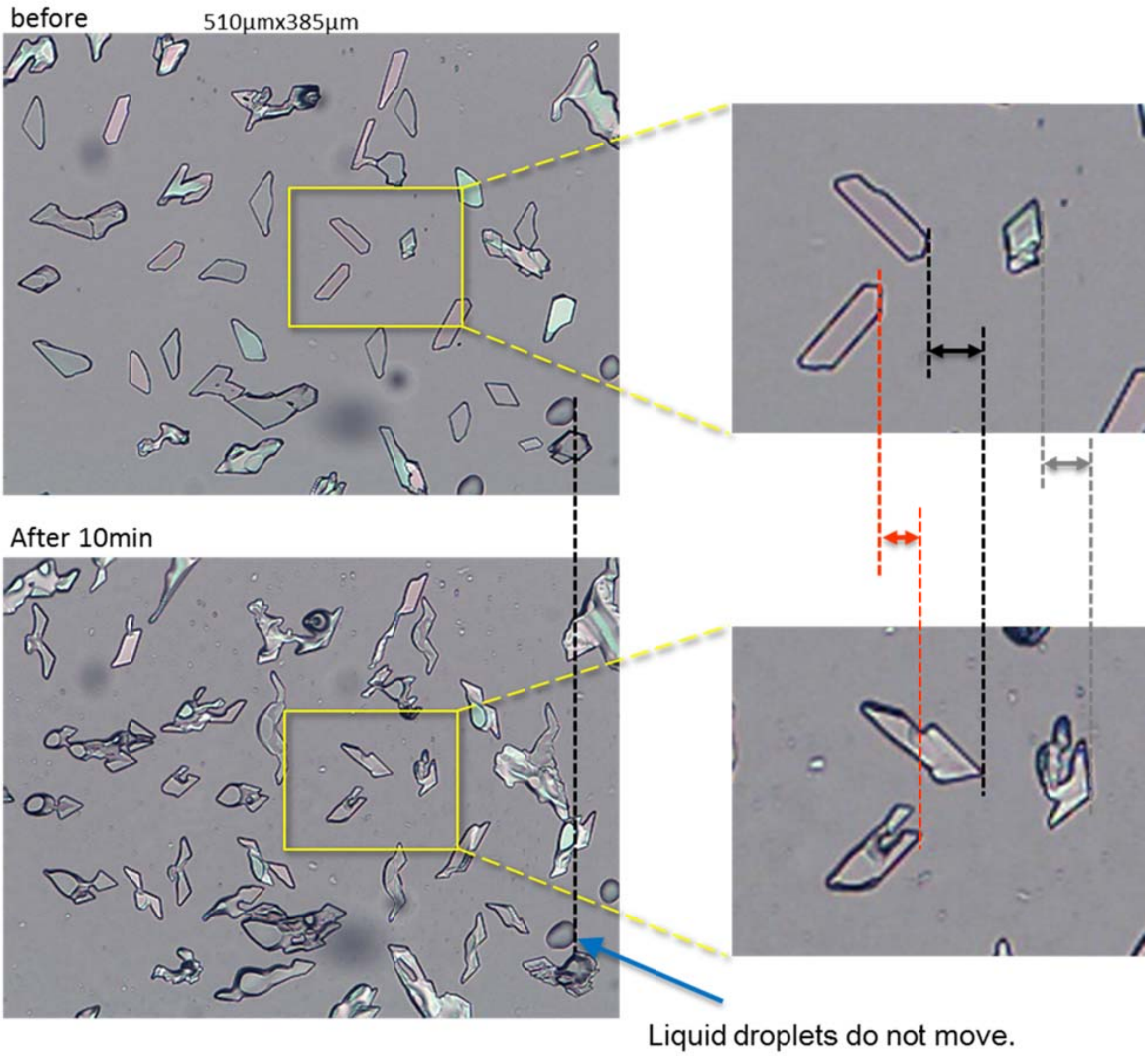

Supplementary Figure 10 Measurement of the distances moved by the crystals.

Supplementary References

1) Yamamoto, S., Nishimura, N. & Hasegawa, S. Steric Effects in Azo Compounds. The Electric Dipole Moments and the Absorption Spectra of Azobenzene Derivatives. *Bull. Chem. Soc. Jpn.* **44,** 2018–2025 (1971).

2) Badger, G., Buttery, R. & Lewis, G. Aromatic azo-compounds. Part I. Oxidation of cis-and trans-azobenzene. *J. Chem. Soc.* 2143–2147 (1953).
